# Coping behavior and risk and resilience stress factors in French regional emergency medicine unit workers: a cross-sectional survey

**Published:** 2016

**Authors:** AI Lala, LM Sturzu, JP Picard, F Druot, F Grama, G Bobirnac

**Affiliations:** * Emergency Medical Service, “Robert PAX” Hospital Center, Sarreguemines, France; ** Psychiatry Department, Lorraine University, Nancy Faculty of Medicine, Nancy, France; *** Surgery Department, Coltea Hospital, Bucharest, Romania; **** “Mina Minovici” National Institute of Legal Medicine, Bucharest, Romania

**Keywords:** emergency department, stress, coping, Brief COPE, Perceived Stress Scale

## Abstract

The Emergency Department (ED) has the highest workload in a hospital, offering care to patients in their most acute state of illness, as well as comforting their families and tending to stressful situations of the physical and psychological areal.

**Method.** A cross-sectional survey of 366 Emergency Unit staff members including medical doctors, medical residents, medical nurses and ward aids, was undergone. Study participants came from four periphery hospitals in the Moselle Department of Eastern France with similar workforce and daily patient loads statistics. The instruments used were the Perceived Stress Scale PSS-10 and the Brief COPE questionnaire.

**Conclusions.** Perceived work overload and overall stress is strongly related to work hours and tend to have a stronger influence on doctors than on the nursing staff. Substance use is a common coping method for medical interns, consistent with prior research. The regular assessment of the ED staff perception of stress and stress related factors is essential to support organizational decisions in order to promote a better work environment and better patient care.

## Introduction

The Emergency Department (ED) has the highest workload in a hospital, offering care to patients in their most acute state of illness, as well as comforting their families and tending to stressful situations of the physical and psychological areal. Working in the emergency department requires a skillset of medical knowledge, psychological robustness, patience, as well as management and coordination aptitudes. 

The stress associated with the ED workers, medical and para-medical alike is generally acknowledged, as well as their increased prevalence of mental health problems, stress and workplace burnout in comparison with the general population [**1**-**3**]. From their training years in their late career, the EM workers suffer from excessive levels of acute and chronic stress and fatigue related to work hours, work-environment factors like emergency department overcrowding and compassion fatigue [**4**,**5**]. 

The quality of life of the EM personnel decreases with age due to above-mentioned factors, leading to slowly developing burnout that may affect their overall health, their levels of job satisfaction and the quality of the services provided [**6**–**9**]. 

The specificity of the emergency medical act strongly manifests itself on account of a wide series of psycho–traumatizing factors augmented both by the vulnerable situation of the patient and the paroxysmal state of the act. Also, it has been recognized that the physical solicitation and distress levels are the highest among all medical specialties. These issues whey on the healthcare workers physical and emotional states and may lead to a reduced capacity and interest in being able to provide the best quality care, to being empathetic towards the patients needs and feelings and can cause a depletion of the ability to cope with the environment and to provide optimal self-care [**10**,**11**]. 

The emergency departments in France suffer from overcrowding, from increased patient intake and poor capacity for patient outtake, the burden of an aging population, the lack of medical personnel, high patient demand standards, and high managerial skill demands on the medical and para-medical staff alike [**12**-**14**]. EM personnel, both medical and para medical, consistently undergo training to surpass the continuously evolving intellectual and psychological necessities in order to assure the best quality of care for their patients. Assessing their risk factors for stress and the ways they cope with it is a key point in establishing strategies to reduce work burnout and mental and physical health issues. 

The goal of this study was to quantify the overall stress values, perceived work overload and perceived self-efficacy, the risk factors that may increase their prevalence and the coping mechanisms engaged. The study addresses all professional fields that act in an emergency department: doctors, medical interns or residents, nurses and the ward aids, as the emergency medical act relies on a constant interaction between these workers. 

Although there is a considerable amount of literature regarding work related burnout and depression, the perceived stress and coping mechanisms are scarcely studied, as well as the risk factors involved. Response to stress has shown to be both gender, age, religion and geographically distributed, as different categories studied show a vast array of generally applied coping mechanisms [**15**,**16**]. While active coping and planning seems to be generally adopted as the principal coping strategies in this field, substance abuse and self blame have been shown to be the more damaging and burnout-generating behavioral mechanisms engaged in order to deal with stress [**17**,**18**]. The importance of stratifying defense mechanisms is crucial, as it is fundamental to implement measures to reduce psychological and physical work field-generated health issues.

## Method 

This study consisted of a cross-sectional survey of 366 Emergency Unit staff members including medical doctors, medical residents, medical nurses, and ward aids. Subject selection was made based on work-field uninterrupted experience of at least one year in an emergency unit. Study participants came from four periphery hospitals in the Moselle Department of Eastern France with similar workforce and daily patient loads statistics. 

Data recovery was made by using Google electronic forms. Vertical survey distribution was made by individual e-mail from the subject’s chief-of-staff of staff secretary explaining the study’s objectives and linking to the form presentation page. The answer to every single question was mandatory for form validation. Data collection was limited to a 10-days period after individual e-mails were sent. 

**Instruments**

The Perceived Stress Scale (PSS-10) 10-unit questionnaire is a bi-dimensional scale analyzing two distinct factors, interpreted in terms of perceived work overload and perceived personal efficacy. Each item is rated on a 5-points scale ranging from never (0) to almost always (4). Scores around 13 are considered average, and high stress groups have a score of around 20 points. Both factors presented good internal consistency and adequate validity of construct in a factorial validation study for the French version [**18**]. The extended analysis showed and illustrated the predicted link between the two factors and the levels of anxiety and depression. 

The French version of the Brief COPE questionnaire is the abridged version of the COPE inventory and presents fourteen scales, all assessing different coping dimensions: active coping, planning, using instrumental support, using emotional support, venting, behavioral disengagement, self-distraction, self-blame, positive reframing, humor, denial, acceptance, religion, and substance use. Each scale contains two items rated 1 to 4, adding up to a total of 28 items. The inventory is built from the theoretical models of Lazarus’ transactional model of stress, and the behavioral self-regulation model of Carver and Scheier. It can be used to assess trait coping (the usual way people cope with stress in everyday life) and state coping (the particular way people cope with a specific stressful situation) [**19**-**21**].

The statistical analysis was made by simple statistics, bivariate and multivariate regression models using equations applied to an Excel spreadsheet and IBM SPSS software. 

## Results

We have issued a total of 366 (N=366) surveys from which we have received a total of 184 (n=184) valid surveys adding to an overall response rate of 50,2%. Professional categories chosen were medical doctors (MD), medical interns (INT), medical nurses (MN) and ward aids (WA). Important response rate value differences were observed across categories (**Table 1**), being higher for the para-medical staff and lower for the medical staff. 

**Table 1:** Response rate variation between professional categories

	sent	received	Response rate
MD	109	42	0,39
INT	62	30	0,48
MN	155	84	0,54
WA	40	28	0,70

The average subject age was 33 years, with no significant differences between gender (33,2 for men and 33,0 for women subjects. 78,8% of the subjects were married or in a couple’s contract union (n=145) while the rest declared to be single or divorced. No significant statistical difference was found between the marital status and gender or professional category. 

**Table 2:** Age and marital status variation between professional categories

	N count	Average age	n Married	n Divorced	n Union	n Single
*INT*	30	27,43	0	0	22 (73%)	8 (27%)
* MD*	42	36,26	16 (38%)	0	22 (52%)	4 (10%)
* MW*	84	33,37	25 (30%)	4	40 (48%)	15 (18%)
* WA*	28	34,14	4 (14%)	0	16 (57%)	4 (14%)

Work hours varied significantly throughout professional subgroups. Medical doctors averaged a 55,2 hours per week (SD=5,00; 48-72), medical interns averaged 62 hours (SD=12; 50-72), including night shifts and overtime, while para medical workers were regulated on a 37 work hour week with very low risk for overtime. 

PSS-10 survey results varied strongly among professional categories, with a higher perceived work overload (PWO) and a lower perceived personal efficacy (PPE) for the medical staff in comparison with the para medical staff. 

Both the medical doctors and the medical interns’ subgroups were correlated with a high risk for stress. Averages PSS-10 scores were 19,00 (SD=2,22; 15-28) for the MD subgroup and 22,22 (SD=5,06; 16-30) for the INT group. 

Medical doctors were not correlated with a high average perceived work overload (r2=1,10; CI=95% 0,93-1,21; z=1,13; p=0,256) and were shown to have a good perceived personal efficacy (r2=1,54; CI=95% 1,02-1,81; z=1,56; p=0,025). On the other hand, the medical intern group showed the strongest correlation with a high perceived work overload (r2=3,02; CI=95% 2,03-4,49; z=5,45; p<0,0001) and a low perceived personal efficacy (r2=3,54; CI=95% 2,02-6,20; z=4,43; p<0,01) of all the studied subgroups. 

Among the para-medical subgroups, ward aids had the lowest global PSS-10 scores averaging 17,84 (SD=3,12; 12-26) while nurses had an average of 17,96 (SD=3,28; 12-26). A higher perceived work overload correlation was found for the MN subgroup (r2=0,74; CI=95% 0,49-1,12; p=0,31) than ward aids (r2=0,62; CI=95% 0,44-1,19; p=0,56), while both professional categories showed lower risks for high stress than the medical subgroups. In complement, both of the subgroups had a higher perceived personal efficacy than the medical professions. 

The average work hours had a powerful influence on the perceived work overload, perceived personal efficacy, and global PSS-10 scores. The analysis showed that the subjects surpassing an average of 37 work hours per week showeda higher perceived work overload (r2=3,44; CI=95% 2,11-5,09; p<0,001) and a lower perceived personal efficacy (r2=3,89; CI=95% 1,91-5,28; p<0,001).

No correlation was found between PSS-10 results and age, marital status or gender statistics. 

Brief COPE results varied between professional subgroups, with a higher tendency of all subgroups towards active coping, acceptance, and planning. Coping methods with the lowest general averages were denial, religion and substance use (**Table 3**).

Medical interns were more prone to substance use and behavioral disengagement than the other subgroups, while medical doctors had a higher average of active coping and instrumental or emotional support.

 No significant differences between age or gender subgroups and coping mechanisms were noticed. 

**Table 3:** Brief COPE results stratified by coping method and professional category subgroup

	Active coping	Planning	Instrumental support	Emotional support	Venting	Positive reinterpretation	Acceptance
General	2,74	2,65	2,62	2,62	2,58	2,75	2,89
MD	3,10	2,89	3,12	3,12	2,88	2,80	3,48
INT	2,57	2,75	2,55	2,55	2,68	2,70	2,97
MN	2,77	2,64	2,55	2,55	2,57	2,81	2,70
MW	2,29	2,21	2,14	2,14	2,07	2,57	2,50
	Denial	Self blame	Humor	Religion	Self distraction	Substance use	Behavioral disengagement
General	1,47	2,50	1,98	1,49	2,61	1,38	1,51
MD	1,31	2,86	2,05	1,38	2,76	1,44	1,51
INT	1,97	2,85	1,88	1,73	2,83	2,13	2,23
MN	1,31	2,23	1,92	1,52	2,58	1,14	1,23
MW	1,64	2,43	2,14	1,29	2,21	1,21	1,57

## Discussion

The results of this study were consistent with the current conclusions of international literature retaining above-average stress levels in the ED physicians and moderate levels in the ED nurses and ward-aids [**1**-**6**]. Higher stress levels were linked with work hours and intern positions. The most prevalent factor accounting for high stress levels was found to be the perceived work overload. This notion is related both to the workload and the perceived time pressure [**22**,**23**] that is a constant and common state in EDs. This suggests that objective measures such as patient load and working hours must be coupled with self-reported measures such as perceived stress and perceived personal efficacy before attempting measures to improve job satisfaction by local managerial efforts [**24**].

Physicians can promote the personal well-being and job satisfaction by assessing decreases in performance related to fatigue and work overload. It is worth noting that although this study found a direct correlation between hours worked and stress levels, workload per-se is defined as the association of hours worked, ED crowding, patient acuity and combined staff skill and interaction quality. Therefore, decisions should be made around balancing these factors while at the same time continuously evaluating their effects on the above-mentioned issues [**25**].

In addendum, factors such as anxiety related to making mistakes, risk of medical malpractice lawsuits and lack of time to evolve via continuous medical education are a constant concern for ED physicians throughout their career. These factors remain controllable at an institutional level. Improving professional autonomy, rewarding compensation and improving collaboration and teamwork in the ED may be ways of improving perception of the working environment as well as improving job-turnover [**26**].

Nursing staff showed better-perceived work overload and personal efficacy, accounting for better overall stress levels than their medical peers. This might be due to the absence of work overtime, improved access to continuous education and better social support from peers [**27**,**29**]. Multiple studies linked longer working hours in the nursing professionals in EDs with a decreased job satisfaction [**27**-**31**], increased levels of stress [**28**,**29**] and burnout [**1**,**28**,**29**] as well as with decreased patient outcomes and patient satisfaction [**30**,**31**]. Although this study did not address all these issues, it is safe to conclude that regulating working hours for nurses increase the chances of improving stress levels and create an environment promoting social support and the access to continuous education and professional improvement.

Coping methods generally engaged by ED workers found in this study were similar to those cited by the international literature. Active coping and planning seem to be the preferred methods of physicians, while nursing staff is prone to active coping, venting and positive reinterpretation. Studies have shown that strategies around venting are associated with high levels of anxiety and depression [**32**]. Nurses seem to be mostly affected by health and safety issues, powerlessness, lack of respect and not feeling valued [**32**], their preferred coping methods addressing these issues respectively, rather than those accounting for stress manifested by doctors. 

The issue of substance use as a coping method among doctors and medical interns has been widely studied [**33**-**35**] and is a finding that is consistent with the results of this study. Higher alcohol use was associated with increased levels of perceived stress, burnout, depression [**34**], and may have consequences for patient safety [**35**].

Since prognosis for recovery of physicians from substance abuse is exceptionally high, managerial and organizational approaches for the early identification of risk behaviors in physicians should be strongly supported [**33**].

**Limitations**

The limitations of this study included the small sample as well as a low response rate, which limited the statistical significance. Also, the geographical selection bias limits called for further and more broad inquiries to enlarge the applicability of the results. The vertical distribution of electronic questionnaires on the work e-mail address may have influenced both the response rate and the subject access and selection, being limited to ED workers who were active on-line at the time of the data gathering. 

## Conclusion

Work related stress is an important issue in the ED working environment. Perceived work overload and overall stress is strongly related to work hours and tendsto have a stronger influence on doctors than on nursing staff. Substance use is a common coping method for medical interns, consistent with prior research. Regular assessment of ED staff perception of stress and stress related factors are essential to support the organizational decisions in order to promote a better work environment and better patient care. 

**Conflicts of interest**

No conflicts of interest are declared. 

**Acknowledgements**

Prof. Raymund Schwan, Psychiatry Department, Lorraine University, Nancy, France

EmanuelleSeris, MD, PhD, Emergency Department, CH Robert PAX, Sarreguemines, France

Prof. Victor Lorin PURCAREA, PhD, Management Department, “Carol Davila” University, Bucarest, Romania

AbdelatifDhifaoui, MD, PhD, Emergency Department, CH Robert PAX, Sarreguemines

Raed Arafat, MD, PhD, SMURD, Romania

## References

[1] Popa F, Arafat R, Purcărea V, Lala A, Bobîrnac G (2010). Occupational Burnout levels in Emergency Medicine – a nationwide study and analysis. Journal of Medicine and Life.

[2] Popa F, Arafat R, Purcărea V, Lală A, Popa–Velea O, Bobîrnac G (2010). Occupational Burnout levels in Emergency Medicine – a stage 2 nationwide study and analysis. Journal of Medicine and Life.

[3] Berwid G (2013). Burnout and Associated Factors among Iranian Emergency Medicine Practitioners. Iranian Journal of Public Health.

[4] Lala A, Bobîrnac G, Tipa R (2010). Stress levels, Alexithymia, Type A and Type C personality patterns in undergraduate students. Journal of Medicine and Life.

[5] Amini A, Munesan MR, Kariman H (2014). Quality of Life in Emergency Medicine Specialists of Teaching Hospitals. Emergency.

[6] Lu DW, Dresden S, McCloskey C, Branzetti J, Gisondi MA (2015). Impact of Burnout on Self-Reported Patient Care Among Emergency Physicians. Western Journal of Emergency Medicine.

[7] Pantenburg B, Luppa M, König HH, Riedel-Heller SG (2016). Burnout among young physicians and its association with physicians’ wishes to leave: results of a survey in Saxony, Germany. Journal of Occupational Medicine and Toxicology.

[8] Wurm W, Vogel K, Holl A (2016). Depression-Burnout Overlap in Physicians. vanWouwe J. PLoS ONE.

[9] Derakhshanfar H, Kashani P, Nassiriabrishamchi S (2015). The Effect of Emergency Department Overcrowding on Efficiency of Emergency Medicine Residents’ Education. Emergency.

[10] Bellolio MF, Cabrera D, Sadosty AT (2014). Compassion Fatigue is Similar in Emergency Medicine Residents Compared to other Medical and Surgical Specialties. Western Journal of Emergency Medicine.

[11] Martini S, Cynthia L (2006). Comparison of Burnout Among Medical Residents Before and After the Implementation of Work Hours Limits. Acad. Psychiatry.

[12] Josland H, Dolan B, Holt L (2008). Stress and stress management. Accident and emergency: theory into practice.

[13] Roessler W (2012). Stress, burnout, and job dissatisfaction in mental health workers. Eur Arch Psychiatry ClinNeurosci.

[14] Durand AC, Gentile S, Gerbeaux P (2011). Be careful with triage in emergency departments: interobserver agreement on 1,578 patients in France. BMC Emergency Medicine.

[15] Gentile S, Vignally P, Durand AC, Gainotti S, Sambuc R, Gerbeaux P (2010). Nonurgent patients in the emergency department?. A French formula to prevent misuse. BMC Health Services Research.

[16] Mason S, Mountain G, Turner J, Arain M, Revue E, Weber EJ (2014). Innovations to reduce demand and crowding in emergency care; a review study. Scandinavian Journal of Trauma, Resuscitation and Emergency Medicine.

[17] Kuo BCH (2014). Coping, acculturation, and psychological adaptation among migrants: a theoretical and empirical review and synthesis of the literature. Health Psychology and Behavioral Medicine.

[18] Rose JS, Campbell M, Skipper G (2014). Prognosis for Emergency Physician with Substance Abuse Recovery: 5-year Outcome Study. Western Journal of Emergency Medicine.

[19] Salmoirago-Blotcher E, Fitchett G, Leung K (2016). An exploration of the role of religion/ spirituality in the promotion of physicians’ wellbeing in Emergency Medicine. Preventive Medicine Reports.

[20] Muller L, Spitz E (2003). Multidimensional assessment of coping: Validation of the Brief COPE among French population. Encephale.

[21] Bellinghausen L, Collange J (2009). Validation factorielle de l’échellefrançaise de stress perçuen milieu professionnel. Santé Publique.

[22] Escriba-Aguir V, Perez-Hoyos S (2007). Psychological well-being and psychosocial work environment characteristics among emergency medical and nursing staff. Stress and Health.

[23] Heyworth J, Whitley TW, Allison EJ Jr., Revicki DA (1993). Correlates of work-related stress among consultants and senior registrars in accident and emergency medicine. Arch. Emerg. Med.

[24] Adriaenssens J, De Gucht V, Maes S (2015). Causes and consequences of occupational stress in emergency nurses, a longitudinal study. J. Nurs. Manag.

[25] Johnston A, Abraham L, Greenslade J (2016). Review article: Staff perception of the emergency department working environment: Integrative review of the literature. Emergency Medicine Australasia.

[26] Healy S, Tyrrell M (2011). Stress in emergency departments: experiences of nurses and doctors. Emerg. Nurse.

[27] Wu H, Sun W, Wang L (2012). Factors associated with occupational stress among Chinese female emergency nurses. Emerg. Med. J.

[28] Adriaenssens J, De Gucht V, Maes S (2015). Causes and consequences of occupational stress in emergency nurses, a longitudinal study. J. Nurs. Manag.

[29] Ross-Adjie G, Leslie G, Gillman L (2007). Occupational stress in the ED: what matters to nurses?. Australas. Emerg. Nurs. J.

[30] Adriaenssens J, De Gucht V, Van Der Doef M, Maes S (2011). Exploring the burden of emergency care: predictors of stress-health outcomes in emergency nurses. J. Adv. Nurs.

[31] Djukic M, Kovner C, Budin WC, Norman R (2010). Physical work environment: Testing an expanded model of job satisfaction in a sample of registered nurses. Nurs. Res.

[32] Kilcoyne M, Dowling M (2007). Working in an overcrowded accident and emergency department: nurses’ narratives. Austral. J. Adv. Nurs.

[33] Oreskovich MR, Shanafelt T, Dyrbye LN, Tan L, Sotile W, Satele D, West CP, Sloan J, Boone S (2015). The prevalence of substance use disorders in American physicians. Am J Addict.

[34] Pedersen AF, Sørensen JK, Bruun NH, Christensen B, Vedsted P (2016). Risky alcohol use in Danish physicians: Associated with alexithymia and burnout?. Drug Alcohol Depend.

[35] Lebensohn P, Dodds S, Benn R, Brooks AJ, Birch M, Cook P, Schneider C, Sroka S, Waxman D, Maizes V (2013). Resident wellness behaviors: relationship to stress, depression, and burnout. Fam Med.

